# Plasma Androgen Receptor and Docetaxel for Metastatic Castration-resistant Prostate Cancer

**DOI:** 10.1016/j.eururo.2018.09.049

**Published:** 2019-03

**Authors:** Vincenza Conteduca, Anuradha Jayaram, Nuria Romero-Laorden, Daniel Wetterskog, Samanta Salvi, Giorgia Gurioli, Emanuela Scarpi, Elena Castro, Mercedes Marin-Aguilera, Cristian Lolli, Giuseppe Schepisi, Antonio Maugeri, Anna Wingate, Alberto Farolfi, Valentina Casadio, Ana Medina, Javier Puente, Mª José Méndez Vidal, Rafael Morales-Barrera, Jose C. Villa-Guzmán, Susana Hernando, Alejo Rodriguez-Vida, Aránzazu González-del-Alba, Begoña Mellado, Enrique Gonzalez-Billalabeitia, David Olmos, Gerhardt Attard, Ugo De Giorgi

**Affiliations:** a Istituto Scientifico Romagnolo per lo Studio e la Cura dei Tumori (IRST) IRCCS, Meldola, Italy; b Centre for Evolution and Cancer, The Institute of Cancer Research, London, UK; c The Royal Marsden NHS Foundation Trust, London, UK; d University College London Cancer Institute, London, UK; e Prostate Cancer Clinical Research Unit, Spanish National Cancer Research Centre, Madrid, Spain; f Hospital Universitario La Princesa, Madrid, Spain; g Hospital Universitario Quirón, Madrid, Spain; h Department of Medical Oncology, IDIBAPS, Hospital Clínico y Provincial, Barcelona, Spain; i Centro Oncológico de Galicia, A Coruña, Spain; j Medical Oncology Department, Hospital Clínico San Carlos, Instituto de Investigación Sanitaria del Hospital Clínico San Carlos (IdISSC), CIBERONC, Madrid, Spain; k Hospital Reina Sofía, Córdoba, Spain; l Vall d’Hebron Institute of Oncology, Vall d’ Hebron University Hospital, Universitat Autònoma de Barcelona, Barcelona, Spain; m Hospital General Universitario de Ciudad Real, Ciudad Real, Spain; n Fundación Hospital Alcorcón, Alcorcón, Spain; o Hospital del Mar, Barcelona, Spain; p Hospital Universitario Son Espases, Palma de Mallorca, Spain; q Department of Hematology & Medical Oncology, Hospital Universitario Morales Meseguer, IMIB-Universidad de Murcia, Murcia, Spain; r Universidad Católica San Antonio de Murcia-UCAM, Murcia, Spain; s CNIO-IBIMA Genitourinary Cancer Research Unit, Hospitales Universitario, virgen de la Victoria y regional de Málaga, Spain

**Keywords:** Castration-resistant prostate cancer, Androgen receptor, Plasma DNA, Docetaxel, Androgen receptor–directed therapies, Biomarker

## Abstract

Plasma androgen receptor (*AR*) gain identifies metastatic castration-resistant prostate cancer (mCRPC) patients with worse outcome on abiraterone/enzalutamide, but its relevance in the context of taxane chemotherapy is unknown. We aimed to evaluate whether docetaxel is active regardless of plasma *AR* and to perform an exploratory analysis to compare docetaxel with abiraterone/enzalutamide. This multi-institutional study was a pooled analysis of *AR* status, determined by droplet digital polymerase chain reaction, on pretreatment plasma samples. We evaluated associations between plasma *AR* and overall/progression-free survival (OS/PFS) and prostate-specific antigen (PSA) response rate in 163 docetaxel-treated patients. OS was significantly shorter in case of *AR* gain (hazard ratio [HR] = 1.61, 95% confidence interval [CI] = 1.08–2.39, *p = *0.018), but not PFS (HR = 1.04, 95% CI 0.74–1.46, *p = *0.8) or PSA response (odds ratio = 1.14, 95% CI = 0.65–1.99, *p *= 0.7). We investigated the interaction between plasma *AR* and treatment type after incorporating updated data from our prior study of 73 chemotherapy-naïve, abiraterone/enzalutamide-treated patients, with data from 115 first-line docetaxel patients. In an exploratory analysis of mCRPC patients receiving first-line therapies, a significant interaction was observed between plasma *AR* and docetaxel versus abiraterone/enzalutamide for OS (HR = 0.16, 95% CI = 0.06–0.46, *p < *0.001) and PFS (HR = 0.31, 95% CI = 0.12–0.80, *p *= 0.02). Specifically, we reported a significant difference for OS favoring abiraterone/enzalutamide for *AR*-normal patients (HR = 1.93, 95% CI = 1.19–3.12, *p = *0.008) and a suggestion favoring docetaxel for *AR*-gained patients (HR = 0.53, 95% CI = 0.24–1.16, *p = *0.11). These data suggest that *AR*-normal patients should receive abiraterone/enzalutamide and *AR*-gained could benefit from docetaxel. This treatment selection merits prospective evaluation in a randomized trial.

**Patient summary:**

We investigated whether plasma androgen receptor (*AR*) predicted outcome in metastatic castration-resistant prostate cancer (mCRPC) patients treated with docetaxel, and we performed an exploratory analysis in patients treated with docetaxel or AR-directed drugs as first-line mCRPC therapy. We showed that plasma *AR* normal favored hormonal treatment, whilst plasma *AR*-gained patients may have had a longer response to docetaxel, suggesting that plasma *AR* status could be a useful treatment selection biomarker.

## Take Home Message

Analysis of metastatic castration-resistant prostate cancer (mCRPC) patients receiving first-line therapy showed that plasma androgen receptor (*AR*)-normal status favored treatment with abiraterone or enzalutamide, whilst docetaxel benefit was unrelated to plasma *AR*. *AR* testing in plasma may have clinical utility for treatment selection in mCRPC.

There are currently several approved life-prolonging therapies for the treatment of metastatic castration-resistant prostate cancer (mCRPC), including androgen receptor (AR)-directed drugs and taxanes. Plasma DNA analysis from mCRPC patients has suggested potential clinical applicability with an association between plasma *AR* aberrations and worse outcome with AR-directed drugs [1], [2], [3], [4], [5]. To date, detection of AR splice variants has been shown to have potential utility for the selection of taxane versus AR-targeted therapy for patients with mCRPC [6], [7]. However, the relevance of plasma *AR* status in the context of taxanes is unknown.

We here aimed to evaluate the association of plasma *AR* status with outcomes in mCRPC patients treated with docetaxel. Additionally, we aimed to perform an exploratory analysis to compare the difference in outcome by plasma *AR* status for patients treated either with first-line docetaxel or AR-directed therapy.

Plasma samples were collected, with the primary aim of biomarker evaluation, from mCRPC patients, treated with standard-dose intravenous docetaxel 75 mg/m^2^ every 3 wk with prednisone 5 mg twice daily for a maximum of 10 cycles for mCRPC [8], between May 2011 and January 2017 in 20 institutions. For the exploratory analysis, we included data on patients from our previous publication [5] who received abiraterone/enzalutamide prior to chemotherapy at the development of mCRPC, with updated clinical follow-up with a cut-off date of December 2017. All patients provided signed consent to an institutional review board-approved protocol before sample collection. Selection criteria, procedures, and the *AR* copy number (CN) droplet digital polymerase chain reaction assay are described in the Supplementary material.

**Fig. 1:**
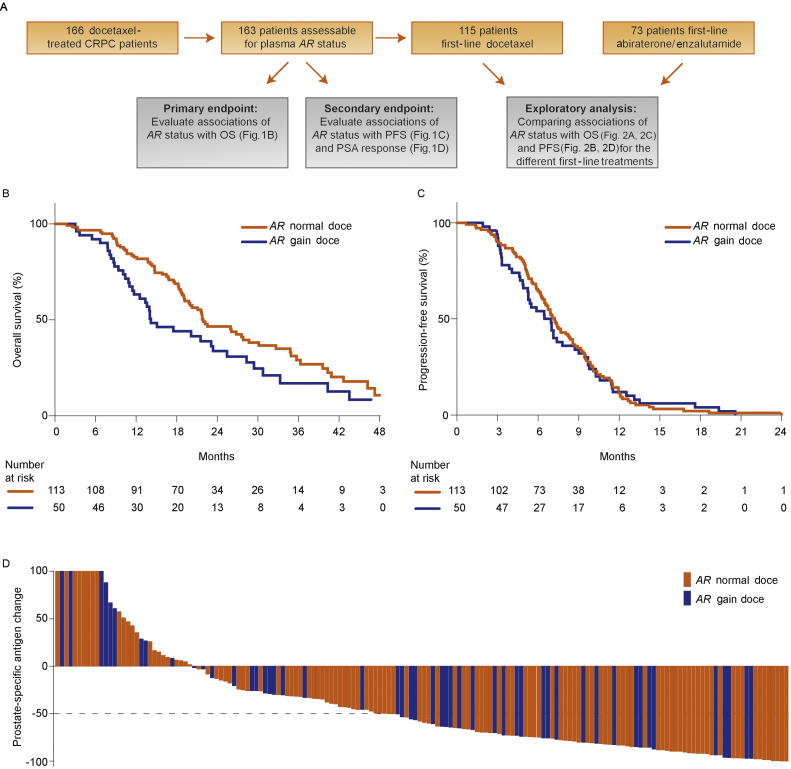
Study design and association of plasma *AR* status with clinical outcome in castration-resistant prostate cancer patients treated with docetaxel. (A) Flow chart showing the selection of docetaxel-treated patients for the primary and exploratory analyses. (B) Overall survival and (C) progression-free survival in docetaxel-treated patients. (D) Waterfall plots depicting prostate-specific antigen (PSA) declines (%) by *AR* copy number normal and gain in docetaxel-treated patients. Bars clipped at maximum 100%. AR = androgen receptor; CN = copy number; CRPC = castration-resistant prostate cancer; doce = docetaxel; OS = overall survival; PFS = progression-free survival.

We set out to determine *AR* status in plasma collected from 166 docetaxel-treated mCRPC patients prior to first- or second-line mCRPC therapy (Fig. 1A), but we had sample failure in three cases. We detected plasma *AR* gain in 50 patients (31%; 28% *AR* gain prior to first-line and 37% prior to second-line therapy). The median number of docetaxel cycles was the same in *AR*-normal and *AR*-gained patients (median 8, interquartile range 6–10). The median follow-up period of alive patients was 24 mo. As 98% of the deaths were prostate cancer related, only overall survival (OS), and not cancer-specific survival, was analyzed. The median OS was 14 mo (95% confidence interval [CI] 12–23) for *AR*-gained patients and 22 mo (95% CI 20–29) for *AR*-normal patients. Median progression-free survival (PFS) was 7 mo (95% CI 5–8) in *AR*-gained patients and 7 mo (95% CI 6–8) in *AR*-normal patients. OS was significantly shorter in *AR*-gained versus *AR*-normal patients (hazard ratio [HR] = 1.61, 95% CI 1.08–2.39, *p *= 0.02), but no significant difference was observed for PFS (HR = 1.04, 95% CI 0.74–1.46,*p* = 0.8) or prostate-specific antigen (PSA) decline ≥50% (odds ratio = 1.14, 95% CI 0.65–1.99, *p *= 0.7; Fig. 1B–D).

Next, we selected the 115 patients treated with docetaxel as first-line therapy and in an exploratory, analysis compared them with 73 previously described patients treated with first-line abiraterone/enzalutamide (Fig. 1A) [5]. A comparison of clinicopathological characteristics between patients receiving either docetaxel or abiraterone/enzalutamide as first-line therapy showed significant differences in age, site of metastases, PSA, lactate dehydrogenase (LDH), hemoglobin, alkaline phosphatase, and plasma *AR* status (Supplementary Table 1). When comparing *AR*-normal with *AR*-gained patients in each treatment group, serum LDH and PSA were significantly higher in *AR*-gained patients treated with abiraterone/enzalutamide and serum alkaline phosphatase was significantly higher in plasma *AR*-gained patients treated with docetaxel (Supplementary Table 2).

The interaction of docetaxel or abiraterone/enzalutamide therapy and *AR* status was investigated using a multivariable Cox proportional hazard model, which showed a significant treatment interaction with *AR* status for both OS (HR = 0.16, 95% CI 0.06–0.46, *p *< 0.001) and PFS (HR = 0.31, 95% CI 0.12–0.80, *p = *0.02; Supplementary Table 3). The median follow-up period for alive patients of the abiraterone/enzalutamide cohort was 32 mo.

**Fig. 2:**
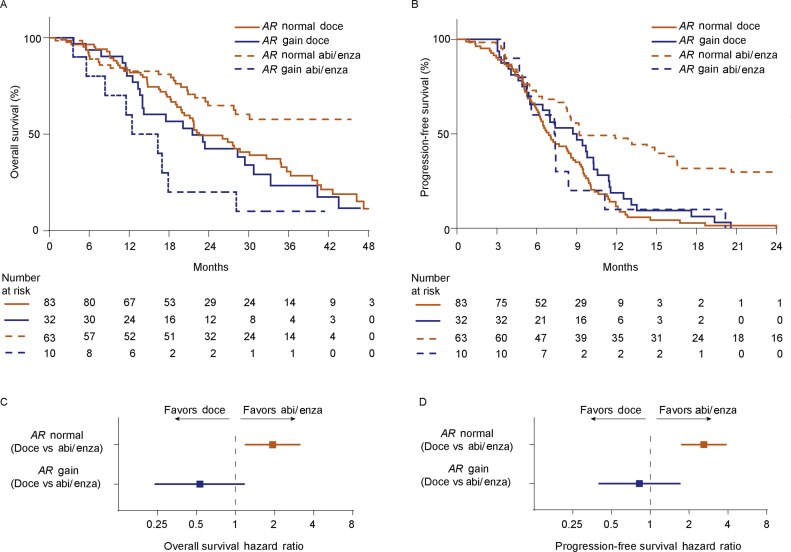
Association of plasma *AR* status with clinical outcome in castration-resistant prostate cancer patients treated with either docetaxel or AR-directed therapies (abiraterone or enzalutamide) as first-line treatment. Interaction between *AR* status and treatment type, after including data from abiraterone- or enzalutamide-treated patients for (A) OS and (B) PFS. (C) Forest plot shows the hazard ratio and 95% confidence interval for (C) OS and (D) PFS in *AR*-normal and *AR*-gained patients. Abi = abiraterone, *AR* *=*  androgen receptor; doce = docetaxel; enza = enzalutamide; OS = overall survival; PFS = progression-free survival.

The estimated median OS and PFS as a function of treatment and *AR* CN status are depicted in Figures 2A and 2B, respectively. The HRs for OS (Fig. 2C) and PFS (Fig. 2D) estimated from the Cox proportional hazard regression analyses suggested that *AR*-normal patients treated with abiraterone/enzalutamide had a significantly lower risk of death (HR = 1.93, 95% CI 1.19–3.12, *p = *0.008) and progressive disease (HR = 2.60, 95% CI 1.75–3.86, *p *< 0.001) when compared with those treated with docetaxel, and in *AR*-gained patients there was a suggestion toward a lower risk of death (HR = 0.53, 95% CI 0.24–1.16, *p = *0.11; Fig. 2C) and progressive disease (HR = 0.82, 95% CI 0.40–1.69, *p = *0.6; Fig. 2D) with docetaxel compared with abiraterone/enzalutamide therapy. In multivariable analysis of first-line treatment patients, including treatment type, plasma *AR* status, and other pretreatment baseline features previously shown to be clinically relevant [5], we observed that plasma *AR* gain was independently associated with worse OS (HR = 6.55, 95% CI 2.74–15.68, *p *< 0.001) and PFS (HR = 3.24, 95% CI 1.47–7.14, *p = *0.004; Supplementary Table 3).

Metastatic CRPC patients can be treated with docetaxel or AR-targeting therapies as first-line therapy. We and others have reported that detection of circulating *AR* aberrations is associated with worse outcome on abiraterone/enzalutamide [2], [3], [4], [5]. In this study, we report that plasma *AR* gain in docetaxel-treated patients was associated with significantly shorter OS. This emphasizes the urgent clinical need of alternative treatments for *AR*-gained patients [9]. Retrospective studies have suggested that AR-V7 expression in mCRPC men can be considered a treatment-specific biomarker associated with superior survival for taxane therapy compared with AR therapies [6], [7]. Here, we explored whether plasma *AR* gain status is associated with resistance to taxanes in an abiraterone/enzalutamide-naïve population and, to avoid the influence of possible cross-resistance events on the interpretation of survival data, we compared it with the effect seen in taxane-naïve abiraterone/enzalutamide-treated patients [10]. The absence of a difference in outcome by *AR* status in treatment-naïve docetaxel-treated patients introduces the hypothesis that *AR*-gained patients would derive greater benefit from treatment with taxanes in preference to abiraterone/enzalutamide. However, we recognize some limitations of our study, including the significantly different durations of median follow-up of alive patients between the docetaxel and the abiraterone/enzalutamide cohort (24 vs 32 mo, with overall follow-up of 24 mo); the relatively modest sample size of the cohorts, especially of *AR*-gained patients treated with abiraterone/enzalutamide (*n *= 10); and the retrospective, nonrandomized design. The majority of patients were treated with taxanes in centers when abiraterone or enzalutamide were not widely available prior to chemotherapy. Nonetheless, there could be a bias due to patient selection, given the different toxicity profiles of taxanes compared with AR-targeting drugs. Additionally, detection of an *AR*-gained clone may be more likely at higher circulating tumor fraction that in itself is prognostic; this could bias the ability to ascertain the predictive value of plasma *AR* with AR-targeting drugs but would not change the interpretation of the absence of difference in our treatment-naive taxane-treated cohort. Lastly, we only considered *AR* gain, but other concurrently assessed AR aberrations (somatic point mutations or splice variants) could provide additional or overlapping information. Our findings suggest that *AR* gain detected in plasma is associated with resistance to abiraterone/enzalutamide but not with taxanes when used in the first-line setting. In conclusion, prospective randomized trials are warranted to validate the utility of plasma *AR* status for treatment selection in mCRPC patients.  

***Author contributions:*** Gerhardt Attard had full access to all the data in the study and takes responsibility for the integrity of the data and the accuracy of the data analysis.  

*Study concept and design*: Conteduca, Jayaram, Wetterskog, Castro, Gonzalez-Billalabeitia, Olmos, Attard, De Giorgi.

*Acquisition of data*: Conteduca, Jayaram, Romero-Laorden, Wetterskog, Salvi, Gurioli, Castro, Wingate, Casadio, Gonzalez-Billalabeitia, Olmos, Attard, De Giorgi.

*Analysis and interpretation of data*: Conteduca, Jayaram, Romero-Laorden, Wetterskog, Castro, Gonzalez-Billalabeitia, Olmos, Attard, De Giorgi.

*Drafting of the manuscript*: Conteduca, Wetterskog, Attard.

*Critical revision of the manuscript for important intellectual content*: All authors.

*Statistical analysis*: Scarpi.

*Obtaining funding*: Gonzalez-Billalabeitia, Olmos, Attard, De Giorgi.

*Administrative, technical, or material support*: Maugeri.

*Supervision*: Attard.

*Other*: Provided patients—Conteduca, Jayaram, Romero-Laorden, Castro, Marin-Aguilera, Lolli, Schepisi, Farolfi, Maugeri, Medina, Puente, Vidal, Morales-Barrera, Villa-Guzmán, Hernando, Rodriguez-Vida, González-del-Alba, Mellado, Gonzalez-Billalabeitia, Olmos, Attard, De Giorgi.  

***Financial disclosures:*** Gerhardt Attard certifies that all conflicts of interest, including specific financial interests and relationships and affiliations relevant to the subject matter or materials discussed in the manuscript (eg, employment/affiliation, grants or funding, consultancies, honoraria, stock ownership or options, expert testimony, royalties, or patents filed, received, or pending), are the following: G. Attard reports receiving commercial research grants from Janssen, Arno Therapeutics, and Innocrin Pharma; has received honoraria and/or travel support from the speakers’ bureaus of Janssen, Astellas, Sanofi-Aventis, and Roche/Ventana; has received travel support from Pfizer, Abbott Laboratories, Bayer Healthcare, and Essa Pharmaceuticals; has ownership interest (including patents) in The Institute of Cancer Research Rewards to Inventors; and is a consultant for/advisory board member of Janssen-Cilag, Veridex, Bayer Healthcare, Roche/Ventana, Astellas, Medivation, Pfizer, Novartis, Millennium Pharma, Abbott Laboratories, and Essa Pharma. V. Conteduca, E. Gonzalez-Billalabeitia, and U. De Giorgi received speaker honoraria or travel support from Astellas, Janssen-Cilag, and Sanofi-Aventis. V. Conteduca received consulting fee from Bayer. D. Olmos received research funding from Janssen and Bayer. J Puente reports receiving commercial research grants from Pfizer and Astellas; has received honoraria and/or travel support from the speakers’ bureaus of Pfizer, Astellas, Janssen, MSD, Roche, Bristol, AstraZeneca, Boehringer, Pierre Fabre, Kyowa, Celgene, Lilly, Merck, Ipsen and Eisai; and is a consultant for Pfizer, Astellas, Janssen, MSD, Bayer, Roche, Bristol, AstraZeneca, Boehringer, Novartis, Clovis, Ipsen, EssaPharma, Eisai and Sanofi. B. Mellado reports receiving commercial research grants from Roche and Bayer; has received travel support from Pfizer and Janssen; and is a consultant for/advisory board member of Roche, Sanofi, Janssen, Astellas Oncology, Pfizer, Novartis, Bristol-Myers Squibb and Ipsen. M. Marín-Aguilera has received travel support from Bristol-Myers Squibb. A. Rodriguez-Vida reports receiving commercial research grants from Takeda, MSD and Pfizer; has received honoraria and/or travel support from the speakers’ bureaus of Janssen, Astellas, Sanofi-Aventis, Bayer, BMS and Roche. N. Romero Laorden has received honoraria and/or travel support from Bayer, Astellas, Janssen-Cilag, and Sanofi-Aventis. M.J. Mendez Vidal has received honoraria and /or travel support from Janssen-Cilag, Bayer Healthcare, Sanofi Aventis, Astellas Medivation, Roche, Novartis and Pfizer. R Morales-Barrera has received honoraria and/or travel support from Bayer, Roche, Astellas, Janssen-Cilag, MSD and Sanofi-Aventis. A. González del Alba has received honoraria and/or travel support from Sanofi Aventis, Astellas Medivation, Janssen-Cilag, Bayer, BMS, Pfizer, Novartis, MSD, Roche, EUSA Pharma and Eisai. E. Castro reports receiving commercial research grants from Astra Zeneca, Bayer, Janssen; has received honoraria and/or travel support from the speakers’ bureaus of Astra Zeneca, Astellas, Bayer, Janssen, Pfizer, Bristol-Myers and Roche; and is an advisory board member of Astellas, Bayer and Janssen. S. Hernando Polo has received honoraria and/or travel support from Sanofi Aventis, Astellas Medivation, Janssen-Cilag, Bayer, BMS, Pfizer, Novartis, MSD and Roche. A. Medina has received honoraria and/or travel support from BMS, Janssen-Cilag, Bayer healthcare, Sanofi Aventis, Astellas Medivation, Roche, Novartis and Pfizer. D. Olmos has a compensated advisory role for Astellas, Astra-Zeneca, Bayer, Clovis, Genetech/Roche, Janssen, and uncompensated advisory role for BioOncotech, Tokai; has received a speaker fee from Astellas, Bayer, Janssen, Sanofi, and travel support from Astellas, Bayer, Janssen, Roche; has received research funding (to the institution): Astra-Zeneca, BioOncotech, Bayer, Janssen. No potential conflicts of interest were disclosed by the other authors.  

***Funding/Support and role of the sponsor*****:** V. Conteduca was funded by a European Society of Medical Oncology Translational Clinical Research Fellowship. A. Jayaram is supported by a grant from the Medical Research Council (MR/P002072/1). G. Attard is supported by a Cancer Research UK Advanced Clinician Scientist Grant (A22744). This work was funded in part by Prostate Cancer UK (PG12-49), the “Instituto de Salud Carlos III” (ISCII) PI16/01565 grant. E. Gonzalez-Billalabeitia was funded by a grant from the “Instituto de Salud Carlos III” (ISCIII) PI15/01499. N. Romero-Laorden was funded by a grant from the “Instituto de Salud Carlos III” (CM14-00200). E. Castro is supported by a Prostate Cancer Foundation Young Investigator Award (2017). E. Castro and D. Olmos are supported by grants from the Ministerio de Economía, Industria y Competitividad (JCI-2014-19129 to E.C., RYC-2015-18625 to D.O.). B. Mellado and M. Marin-Aguilera work were supported by the Instituto de Salud Carlos III-Subdirección General de Evaluación y Fomento de la Investigación (PI12/01226 and PI15/676) and co-funded by the European Regional Development Fund. Funding from CERCA Programme/Generalitat de Catalunya is gratefully acknowledged. During the conduct of the study, E. Castro was supported by a grant from the Ministerio de Educación, Cultura y Deportes (CAS17/00182). The funders of the study had no role in study design, data collection, data analysis, data interpretation, or writing of the report. The corresponding authors had full access to all data and had the final responsibility for the decision to submit for publication.  

***Acknowledgments:*** We thank the participating men and their families who suffered from metastatic prostate cancer and nonetheless gave the gift of participation so that others might benefit.
