# Supplementary material for: Plasma Androgen Receptor and Docetaxel for Metastatic Castration-resistant Prostate Cancer
Source: Eur Urol. 2019 Mar;75(3):368–73. doi: 10.1016/j.eururo.2018.09.049 (PMC6377278; doi:10.1016/j.eururo.2018.09.049)
Supplement: Supplementary file 1 [file mmc1.doc]

**Supplementary material**

**Supplementary methods**

**1. Eligibility criteria**

***1.1. Inclusion criteria***

1. Patients to have histologically confirmed adenocarcinoma of the prostate without neuroendocrine differentiation or small cell histology

2. Patients having progressive disease (PD) despite “castration levels” of serum testosterone (<50 ng/dl; ≤1.73 nmol/l), and ongoing LHRH analog treatment or prior surgical castration

3. Progression as defined by at least two of the following: a rise in prostate-specific antigen (PSA), worsening symptoms, or radiological progression, namely, progression in soft tissue lesions measured by computed tomography (CT) imaging according to the modified Response Evaluation Criteria in Solid Tumors (RECIST) or progression on bone scanning according to the criteria adapted from the Prostate Cancer Working Group (PCWG3) criteria.

4. Patients not having received radiotherapy, chemotherapy, or immunotherapy at least 30 d prior to the treatment

5. Male, aged ≥18 yr.

6. Life expectancy >3 mo

7. Eastern Cooperative Oncology Group performance status ≤2

8. Patients willing to use a method of birth control with adequate barrier protection

9. Patients to have normal organ and marrow function as defined below:

(a) Leukocytes >3000/ml

(b) Absolute neutrophil count >1500/ml

(c) Platelets >100 000/ml

(d) Total bilirubin within normal institutional limits

(e) AST(SGOT)/ALT(SGPT) <2.5 × institutional upper limit of normal

(f) Creatinine within normal institutional limits

10. No evidence (within 5 yr) of prior malignancies (except for successfully treated basal cell or squamous cell carcinoma of the skin)

11. Participants willing and able to give informed consent for participation in the study

***1.2. Exclusion criteria***

1. Patients who have had previous therapy with abiraterone and/or enzalutamide

2. Patients starting therapy with no standard dose of docetaxel

3. Concurrent use of other anticancer agents or treatments, with the following exceptions:

(a) LHRH agonists or antagonists

(b) Denosumab or bisphosphonate (eg, zoledronic acid)

3. Uncontrolled intercurrent illness including, but not limited to, ongoing or active infection, symptomatic congestive heart failure, unstable angina pectoris, cardiac arrhythmia, or psychiatric illness/social situations that would limit compliance with study requirements

5. Having known allergies, hypersensitivity, or intolerance to docetaxel, prednisone, or their excipients

8. Other primary tumor (other than castration-resistant prostate cancer) including hematological malignancy present within the last 5 yr (except nonmelanoma skin cancer or low-grade superficial bladder cancer)

**2. Procedures**

Serum PSA, serum lactate dehydrogenase, alkaline phosphatase, and blood cell count were assessed within 1 wk of starting treatment and before every therapy cycle thereafter. Documentation of PD was considered radiographic evidence of new lesions by bone scintigraphy, and/or new or enlarging soft tissue lesions by CT or magnetic resonance imaging, according to the Prostate Cancer Clinical Trials Working Group 3 (PCWG3) guidelines [1]. We used CT and bone scan at the time of screening and every 12 wk on treatment. However, in clinical practice, deterioration in clinical conditions and/or radiological progression according to local radiologist’s evaluation was also a criterion sufficient to establish PD and discontinuation of treatment.

**3. Detection of *AR* aberrations by digital droplet polymerase chain reaction in plasma samples**

Peripheral blood samples were collected within 30 d of treatment initiation, drawn into 10-ml tubes with anticoagulant, maintained at room temperature, processed within 30 min, and stored at –80°C. Circulating DNA was extracted from 1 to 2 ml of plasma with the QIAamp Circulating Nucleic Acid Kit (Qiagen) and quantified with the Quant-iT high sensitivity PicoGreen double-stranded DNA Assay Kit (Invitrogen) or by spectrophotometric evaluation (NanoDrop ND-1000; Celbio, Milan, Italy).

We performed a multiplex digital droplet polymerase chain reaction (PCR) at the Institute of Cancer Research, Sutton, UK, using assay [2] for the evaluation of *AR* copy number, using three reference genes (*NSUN3*, *ElF2C1*, and *AP3B1*) and *ZXDB* at Xp11.21 as a control gene not involving the whole arm of chromosome. Each PCR reaction was prepared with 1–2 ng DNA, 10 μl 2 × Supermix and a total volume of primer probe assays of 2 μl in a total volume of 20 μl. PCR reactions were partitioned into ~20 000 droplets per sample with an automated droplet generator (Bio-Rad). Emulsified PCR reactions were run on a Mastercycler Nexus GSX1 (Eppendorf). Digital PCR analysis was performed with QuantaSoft v1.3.2.0 software to evaluate the number of positive droplets. At least two negative control wells with no DNA and positive control wells with a known *AR* copy number were included in every run.

**4. Statistical analysis**

In this study, overall survival (OS) was calculated from the start of docetaxel therapy until death or last follow-up. Patients still alive at time of last follow-up were censored. Progression-free survival (PFS) was calculated from the 1st day of therapy to the date of progression of disease or death from any cause, or the date of last tumor evaluation. Radiographic progression was defined using Response Evaluation Criteria in Solid Tumors version 1.1. PSA decline was evaluated according to PCWG3 guidelines [1].

Data were summarized by frequency for categorical variables and by median and range for continuous variables. Association between categorical variables was assessed using the chi-square test or the Fisher’s exact test, as appropriate, or the median test (for continuous variables).

Survival curves were estimated by the Kaplan-Meier method and were compared using the log-rank test. Univariate and multivariable Cox regression models were used to investigate potential predictors of PFS and OS, and to estimate hazard ratios and their 95% confidence intervals. Odds ratios of PSA response were assessed using a logistic regression model.

We then incorporated updated data on OS and PFS from our prior study of docetaxel-naive patients treated with abiraterone or enzalutamide to compare the impact of plasma *AR* copy number in the context of docetaxel versus *AR*-directed therapy. The interaction of therapy and *AR* status was also investigated using a multivariable Cox proportional hazards model.

All *p* values were two sided, and *p* < 0.05 was considered statistically significant. Statistical analyses were performed with SAS 9.4 software (SAS Institute, Cary, NC, USA).

**References**

1. Scher HI, Morris MJ, Stadler WM, et al. Trial design and objectives for castration-resistant prostate cancer: updated recommendations from the Prostate Cancer Clinical Trials Working Group 3. J Clin Oncol 2016;34:1402–18.
2. Conteduca V, Wetterskog D, Sharabiani MTA, et al. Androgen receptor gene status in plasma DNA associates with worse outcome on enzalutamide or abiraterone for castration-resistant prostate cancer: a multi-institution correlative biomarker study. Ann Oncol 2017;28:1508–16.

**Supplementary Table 1 – Comparison of baseline characteristics of first-line patients treated with docetaxel (abiraterone or enzalutamide naïve) and patients treated with abiraterone or enzalutamide (chemotherapy** naïve)

|  | Docetaxel  (*n* = 115) | Abi or enza  (*n* = 73) | *p* value |
| --- | --- | --- | --- |
| Age (yr), median (IQR) | 70 (65–75) | 73 (69–82) | 0.04 |
| ECOG performance status, *n* (%) | |  |  |
| 0–1 | 99 (87) | 69 (94) |  |
| ≥2 | 15 (13) | 4 (6) |  |
| Unknown/missing | 1 | 0 | 0.09 |
| Gleason score, *n* (%) |  |  |  |
| <8 | 34 (32) | 36 (51) |  |
| ≥8 | 73 (68) | 34 (49) |  |
| Unknown/missing | 8 | 3 | 0.009 |
| Bone metastases, *n* (%) |  |  |  |
| No | 14 (12) | 29 (40) |  |
| Yes | 101 (88) | 44 (60) | <0.001 |
| Visceral metastases, *n* (%) |  |  |  |
| No | 90 (78) | 70 (96) |  |
| Yes | 25 (22) | 3 (4) | 0.001 |
| Liver metastases, *n* (%) |  |  |  |
| No | 106 (92) | 72 (99) |  |
| Yes | 9 (8) | 1 (1) | 0.09 |
| Nodal metastases**,** *n* (%) |  |  |  |
| No | 62 (54) | 44 (60) |  |
| Yes | 52 (46) | 29 (40) | 0.4 |
| Serum PSA (mg/l), median (IQR) | 49.7 (14.4–143) | 32 (10.2–81.4) | 0.04 |
| Serum LDH (U/l), *n* (%) |  |  |  |
| <225 | 52 (48) | 67 (92) |  |
| ≥225 ^a^ | 57 (52) | 6 (8) |  |
| Unknown/missing | 6 | 0 | <0.001 |
| Hemoglobin (g/l), *n* (%) |  |  |  |
| <12.5 | 43 (37) | 7 (10) |  |
| ≥12.5 ^a^ | 72 (63) | 66 (90) | <0.001 |
| Unknown/missing |  |  |  |
| ALP (U/l), *n* (%) |  |  |  |
| <129 | 38 (36) | 58 (81) |  |
| ≥129 ^a^ | 68 (64) | 14 (19) |  |
| Unknown/missing | 9 | 1 | <0.001 |
| Plasma *AR* status, *n* (%) |  |  |  |
| Normal | 83 (72) | 63 (86) |  |
| Gain b | 32 (28) | 10 (14) | 0.02 |
| Median follow-up (mo), (IQR) | 24 (18–36) | 32 (25–39) | 0.003 |
| Death events, *n* | 79 | 33 | 0.001 |
| Median OS (mo), (95% CI) | 23 (20–29) | NR | 0.02 |
| Median PFS (mo), (95% CI) | 7 (6–9) | 13 (8–17) | <0.001 |

Abi = abiraterone; ALP = alkaline phosphatase; *AR* = androgen receptor; CI = confidence interval; ECOG = Eastern Cooperative Oncology Group; enza = enzalutamide; IQR = interquartile range; LDH = lactate dehydrogenase; *n* = number; NR = not reached; OS = overall survival; PFS = progression-free survival; PSA = prostate-specific antigen.

^a^ Upper normal value.

^b^ *AR* copy number cut-point of 2.01 assessed by using maximum log likelihood as correlative statistics in a multivariable Cox regression model by an approach described previously [8].

**Supplementary Table 2 – Baseline characteristics of first-line docetaxel-treated patients and abiraterone- or enzalutamide-treated patients based on plasma *AR* status**

|  | Docetaxel  (*n* = 115) | | |  | | |  | | Abi or enza  (*n* = 73) | | | | | | | | | |  |  | | | |  |
| --- | --- | --- | --- | --- | --- | --- | --- | --- | --- | --- | --- | --- | --- | --- | --- | --- | --- | --- | --- | --- | --- | --- | --- | --- |
|  | *AR* normal  (*n* = 83) | *AR* gain  (*n* = 32) | | | *p* value | | | |  | | *AR n*ormal  (*n* = 63) | | | | *AR* gain  (*n* = 10) | *p* value | | | | | | |  | |
| Age (yr), median (IQR) | 70 (65–75) | 71 (64–74) | | | 0.7 | | | | |  | | 73 (69–82) | | 71 (71–78) | | | | 0.4 | | | |  | | |
| ECOG performance status, *n* (%) | | |  | | |  | | | |  | |  | |  | | | |  | | | |  | | |
| 0–1 | 73 (88) | 26 (84) | | |  | | | | |  | | 59 (94) | | 10 (100) | | | |  | | | |  | | |
| ≥2 | 10 (12) | 5 (16) | | |  | | | | |  | | 4 (6) | | 0 | | | |  | | | |  | | |
| Unknown/missing | 0 | 1 | | | 0.5 | | | | |  | | 0 | | 0 | | | | 0.4 | | | |  | | |
| Gleason score, *n* (%) |  |  | | |  | | | | |  | |  | |  | | | |  | | | |  | | |
| <8 | 22 (29) | 12 (40) | | |  | | | | |  | | 33 (55) | | 3 (30) | | | |  | | | |  | | |
| ≥8 | 55 (71) | 18 (60) | | |  | | | | |  | | 27 (45) | | 7 (70) | | | |  | | | |  | | |
| Unknown/missing | 6 | 2 | | | 0.3 | | | | |  | | 3 | | 0 | | | | 0.2 | | | |  | | |
| Bone metastases, *n* (%) |  |  | | |  | | | | |  | |  | |  | | | |  | | | |  | | |
| No | 11 (13) | 3 (9) | | |  | | |  | | 26 (41) | | | 3 (30) | | | |  | | | |  | | | |
| Yes | 72 (87) | 29 (91) | | | 0.7 | | | | |  | | 37 (59) | | 7 (70) | | | | 0.7 | | | |  | | |
| Visceral metastases, *n* (%) |  |  | | |  | | | | |  | |  | |  | | | |  | | | |  | | |
| No | 66 (79) | 24 (75) | | |  | | | | |  | | 60 (95) | | 10 (100) | | | |  | | | |  | | |
| Yes | 17 (21) | 8 (25) | | | 0.6 | | | | |  | | 3 (5) | | 0 | | | | 0.5 | | | |  | | |
| Liver metastases, *n* (%) |  |  | | |  | | | | |  | |  | |  | | | |  | | | |  | | |
| No | 77 (93) | 29 (91) | | |  | | | | |  | | 62 (98) | | 10 (100) | | | |  | | | |  | | |
| Yes | 6 (7) | 3 (9) | | | 0.7 | | | | |  | | 1 (2) | | 0 | | | | 0.7 | | | |  | | |
| Nodal metastases, *n* (%) |  |  | | |  | | | | |  | |  | |  | | | |  | | | |  | | |
| No | 47 (57) | 15 (47) | | |  | | | | |  | | 39 (62) | | 5 (50) | | | |  | | | |  | | |
| Yes | 35 (43) | 17 (53) | | | 0.3 | | | | |  | | 24 (38) | | 5 (50) | | | | 0.5 | | | |  | | |
| Serum PSA (mg/l), median (IQR) | 42.9 (14.4–123) | 99 (18.9–223) | | | 0.1 | | | | |  | | 20.4 (6.6–58.5) | | 137.0 (104–298) | | | | <0.001 | | | |  | | |
| Serum LDH (U/l), *n* (%) |  |  | | |  | | | | |  | |  | |  | | | |  | | | |  | | |
| <225 | 36 (47) | 16 (50) | | |  | | | | |  | | 60 (95) | | 7 (70) | | | |  | | | |  | | |
| ≥225 ^a^ | 41 (53) | 16 (50) | | |  | | | | |  | | 3 (5) | | 3 (30) | | | |  | | | |  | | |
| Unknown/missing | 6 | 0 | | | 0.8 | | | | |  | | 0 | | 0 | | | | 0.03 | | | |  | | |
| Hemoglobin (g/dl), *n* (%) |  |  | | |  | | | | |  | |  | |  | | | |  | | | |  | | |
| <12.5 | 28 (34) | 15 (47) | | |  | | | | |  | | 6 (10) | | 1 (10) | | | |  | | | |  | | |
| ≥12.5 ^a^ | 55 (66) | 17 (53) | | | 0.2 | | | | |  | | 57 (90) | | 9 (90) | | | | 0.9 | | | |  | | |
| ALP (U/l), *n* (%) |  |  | | |  | | | | |  | |  | |  | | | |  | | | |  | | |
| <129 | 33 (44) | 5 (16) | | |  | | | | |  | | 53 (84) | | 5 (56) | | | |  | | | |  | | |
| ≥129 ^a^ | 42 (56) | 26 (94) | | |  | | | | |  | | 10 (16) | | 4 (44) | | | |  | | | |  | | |
| Unknown/missing | 8 | 1 | | | 0.007 | | | | |  | | 0 | | 1 | | | | 0.06 | | | |  | | |
| Clinical survival data |  |  | | |  | | | | |  | |  | |  | | | |  | | | |  | | |
| Median follow-up (mo), (IQR) | 23 (19–38) | 27 (16–33) | | | 0.8 | | | | |  | | 32 (24–39) | | 41 (4–41) | | | | 0.4 | | | |  | | |
| Death events, *n* | 56 | 23 | | | 0.7 | | | | |  | | 24 | | 9 | | | | 0.004 | | | |  | | |
| Median OS (mo), (95% CI) | 23 (20–33) | 21 (14–31) | | | 0.5 | | | | |  | | NR | | 14 (4–18) | | | | <0.001 | | | |  | | |
| Median PFS (mo), (95% CI) | 7 (6–9) | 9 (5–10) | | | 0.2 | | | | |  | | 15 (9–17) | | 7 (4–8) | | | | 0.01 | | | |  | | |

Abi = abiraterone; ALP = alkaline phosphatase; *AR* = androgen receptor; CI = confidence interval; CN = copy number; ECOG = Eastern Cooperative Oncology Group; enza = enzalutamide; IQR = interquartile range; LDH = lactate dehydrogenase; *n* = number; NR = not reached; OS = overall survival; PFS = progression-free survival; PSA = prostate-specific antigen.

^a^ Upper normal value.

^b^ AR CN cut-point of 2.01 assessed by using maximum log likelihood as correlative statistics in a multivariable Cox regression model by an approach described previously [8].

**Supplementary Table 3 – Multivariable analysis of OS and PFS in first-line patients**

|  | OS | |  | PFS | | |  |
| --- | --- | --- | --- | --- | --- | --- | --- |
|  | HR (95% CI) | *p* value | HR (95% CI) | | *p* value |  | |
| Age (continuous variable) (n = 164) | 1.01 (0.98–1.04) | 0.5 | 0.99 (0.96–1.01) | | 0.2 |  | |
| ECOG performance status |  |  |  | |  |  | |
| 0–1 (n = 146) | 1.00 |  | 1.00 | |  |  | |
| ≥2 (n = 18) | 1.98 (0.99–3.96) | 0.05 | 2.52 (1.41–4.51) | | 0.002 |  | |
| Gleason score |  |  |  | |  |  | |
| <8 (n = 63) | 1.00 |  | 1.00 | |  |  | |
| ≥8 (n = 101) | 0.86 (0.54–1.37) | 0.5 | 1.09 (0.75–1.59) | | 0.7 |  | |
| Bone metastases |  |  |  | |  |  | |
| No (n = 40) | 1.00 |  | 1.00 | |  |  | |
| Yes (n = 124) | 1.38 (0.79–2.43) | 0.3 | 1.13 (0.72–1.76) | | 0.6 |  | |
| Visceral metastases |  |  |  | |  |  | |
| No (n = 142) | 1.00 |  | 1.00 | |  |  | |
| Yes (n = 22) | 0.90 (0.42–1.95) | 0.8 | 0.74 (0.40–1.36) | | 0.3 |  | |
| Liver metastases |  |  |  | |  |  | |
| No (n = 157) | 1.00 |  | 1.00 | |  |  | |
| Yes (n = 7) | 3.17 (1.01–9.91) | 0.05 | 2.05 (0.79–5.29) | | 0.14 |  | |
| Nodal metastases |  |  |  | |  |  | |
| No (n = 95) | 1.00 |  | 1.00 | |  |  | |
| Yes (n = 69) | 1.07 (0.68–1.68) | 0.8 | 1.21 (0.85–1.74) | | 0.3 |  | |
| Baseline PSA (mg/l;  continuous variable) (n = 164) | 1.00 (1.00–1.01) | 0.2 | 1.00 (0.99–1.01) | | 0.8 |  | |
| LDH (U/l) |  |  |  | |  |  | |
| <225 (n = 109) | 1.00 |  | 1.00 | |  |  | |
| ≥225 (n = 55) | 1.37 (0.83–2.26) | 0.2 | 1.43 (0.92–2.21) | | 0.11 |  | |
| Hemoglobin (g/dl) |  |  |  | |  |  | |
| ≥12.5 (n = 122) | 1.00 |  | 1.00 | |  |  | |
| <12.5 (n = 42) | 1.10 (0.67–1.80) | 0.7 | 0.71 (0.46–1.08) | | 0.11 |  | |
| ALP (U/l) |  |  |  | |  |  | |
| <129 (n = 88) | 1.00 |  | 1.00 | |  |  | |
| ≥129 (n = 76) | 1.54 (0.92–2.59) | 0.10 | 1.21 (0.81–1.81) | | 0.3 |  | |
| *Plasma AR* status |  |  |  | |  |  | |
| Normal (n = 127) | 1.00 |  | 1.00 | |  |  | |
| Gain (n = 37) | 6.55 (2.74–15.68) | <0.001 | 3.24 (1.47–7.14) | | 0.004 |  | |
| Therapy |  |  |  | |  |  | |
| Abi/enza (n = 69) | 1.00 |  | 1.00 | |  |  | |
| Docetaxel (n = 95) | 1.07 (0.58–1.95) | 0.8 | 2.37 (1.39–4.04) | | 0.002 |  | |
| Plasma *AR* status therapy interaction  (n = 164) | 0.16 (0.06–0.46) | <0.001 | 0.31 (0.12–0.80) | | 0.02 |  | |

This table includes patients treated with docetaxel or AR-directed drugs as first-line therapy with data available for all variables (n = 164, Supp Table 1)

ALP = alkaline phosphatase; *AR* = androgen receptor; CI = confidence interval; ECOG = Eastern Cooperative Oncology Group; HR = hazard ratio; LDH = lactate dehydrogenase; OS = overall survival; PFS = progression-free survival; PSA = prostate-specific antigen.
